# A molecular toolkit to boost functional genomic studies in transformation-recalcitrant vegetable legumes

**DOI:** 10.1093/hr/uhad064

**Published:** 2023-04-10

**Authors:** Xinyang Wu, Peipei Zhang, Shuting Chen, Zixin Zhang, Yihan Zhang, Pingping Fang, Kang Ning, Ting Sun, Pei Xu

**Affiliations:** Key Laboratory of Specialty Agri-Product Quality and Hazard Controlling Technology of Zhejiang, College of Life Sciences, China Jiliang University, Hangzhou, China; Zhejiang Lab, Hangzhou, China; Key Laboratory of Specialty Agri-Product Quality and Hazard Controlling Technology of Zhejiang, College of Life Sciences, China Jiliang University, Hangzhou, China; Key Laboratory of Specialty Agri-Product Quality and Hazard Controlling Technology of Zhejiang, College of Life Sciences, China Jiliang University, Hangzhou, China; Key Laboratory of Specialty Agri-Product Quality and Hazard Controlling Technology of Zhejiang, College of Life Sciences, China Jiliang University, Hangzhou, China; Key Laboratory of Specialty Agri-Product Quality and Hazard Controlling Technology of Zhejiang, College of Life Sciences, China Jiliang University, Hangzhou, China; Key Laboratory of Specialty Agri-Product Quality and Hazard Controlling Technology of Zhejiang, College of Life Sciences, China Jiliang University, Hangzhou, China; Key Laboratory of Specialty Agri-Product Quality and Hazard Controlling Technology of Zhejiang, College of Life Sciences, China Jiliang University, Hangzhou, China; Key Laboratory of Specialty Agri-Product Quality and Hazard Controlling Technology of Zhejiang, College of Life Sciences, China Jiliang University, Hangzhou, China

Dear Editor

Legumes, the second-largest family of crops, contribute over one-third of human dietary proteins. Soybean (*Glycine max* L*.*), common bean (*Phaseolus vulgaris* L*.*), pea (*Pisum sativum* L.), and cowpea (*Vigna unguiculata* L.) are among the most widely cultivated crop legumes for grain and vegetable and are essential for food security globally. Their reference genomes have been decoded but, with the only exception of soybean, their functional genomics have lagged far behind genome assembly due to the transformation-recalcitrant nature of these species [1]. Hairy root transformation has been well established, but its usefulness in gene function investigation in aerial organs is limited. Virus-induced gene silencing (VIGS) has also found applications, but the most widely distributed bean pod mosaic virus-based system relies on the costly biolistic method [2]; other systems, like pea early browning virus-based VIGS, have not been proved for their universality. Genetic and omics studies have identified numerous quantitative trait loci (QTLs)/candidate genes governing various agriculturally important traits [1]. This necessitates the development of efficient and reproducible research tools for verification of gene function.

**Figure 1:**
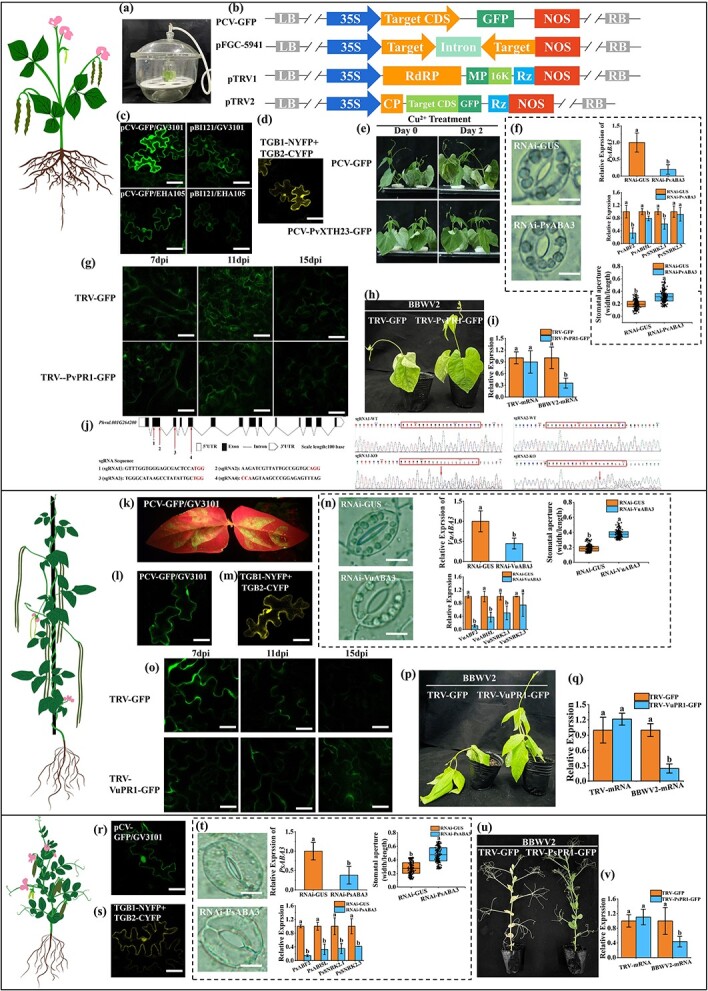
The developed molecular toolkit for legume crops. **a** Negative pressure vacuum pumping for *Agrobacterium* strain infiltration. **b** Constructed vectors used in this study. **c**, **k**, **l**, **r** GFP fluorescence images of *in planta* transient overexpression; **c** shows confocal microscopy images of common bean leaves infiltrated with two *Agrobacterium* strains (GV3101 and EHA105) carrying the PCV-GFP or PBI121-GFP construct; **k** is a GFP image of infiltrated cowpea leaves under a fluorescence lamp; **l** and **r** are confocal microscopy images of cowpea and pea leaves infiltrated with PCV-GFP. **d**, **m**, **s** BiFC images of TGB1 and TGB2 interaction in common bean, cowpea, and pea leaves, respectively. **e** Phenotypes of transiently expressed common bean seedlings before and after CuCl_2_ treatment. Pictures are reproduced from Fang *et al*. [4]. **f**, **n**, **t** Representative images of stomata (scale bars = 10 μm) from the control and *ABA3*-silenced common bean, cowpea, and pea plants, respectively, and the relative expression of *ABA3* and ABA pathway downstream genes as well as stomatal apertures. **g**, **o** Confocal microscopy images of TRV-GFP- and TRV-PR1-GFP-infiltrated common bean and cowpea cells at 7, 11, and 15 dpi. **h**, **p**, **u** Phenotypes of TRV-GFP- and TRV-PR1-GFP-infiltrated common bean, cowpea, and pea plants inoculated with the pathogen BBWV2. 1-week-old common bean and cowpea plants were infiltrated with *Agrobacterium*, inoculated with BBWV2 at 7 dpi and imaged at 14 dpi. 2-week-old pea plants were infiltrated with *Agrobacterium*, inoculated with BBWV2 at 7 dpi and imaged at 21 dpi. **i**, **q**, **v** Levels of TRV and BBWV2 mRNA in leaves of TRV-GFP- and TRV-PR1-GFP-infiltrated common bean, cowpea, and pea plants. **j** Structure diagram, *PvPDS* sgRNA target positions and sequences, and the sequences of edited and non-edited *PvPDS* gene. Different letters in histograms indicate significant differences (*P* < .05). Unless specified, scale bars = 50 μm.

**Table 1:** List of gene IDs and primers used in the study.

Gene ID	Primer name	Sequence 5′–3′
Phvul.006G197400	PvPR1-F	ATGGGGTTGTGCATTAAGGT
	PvPR1-R	GTAGGGTCTTTCGCCAAGAA
Phvul.011G216100	PvABA3-RNAi-BamHI-F	TGGAGAGGACACGCGGGATCCAGTTAGAGAAGAGAGGATTTTGAAGCC
	PvABA3-RNAi-BamHI-R	GGCGCGCCCCATGCGGGATCCGCAAAGGGATGTGCTACTGTGC
	PvABA3-RNAi-XbaI-F	TTGCAGGTATTTGGCTCTAGAGCAAAGGGATGTGCTACTGTGC
	PvABA3-RNAi-XbaI-R	GGTCTTAATTAACTCTCTAGAAGTTAGAGAAGAGAGGATTTTGAAGCC
	PvABA3-RT-F	TGCAAGTAGGTTTCGTCCCA
	PvABA3-RT-R	GCCCCCTAGCGAACTGAAAT
Phvul.003G291800	PvABF2-RT-F	TTTGGGTCCATGAACATGGA
	PvABF2-RT-R	CTTCCAAACCTCATCCACTGT
Phvul.005G011600	PvABI5L-RT-F	GAAGACTGTGGATGAGGTTTG
	PvABI5L-RT-R	CACCAAGAAATCCTCCAATGTC
Phvul.009G157800	PvSnRK2.1-RT-F	GTTACAAGGAGGTGGTTTTGAC
	PvSnRK2.1-RT-R	AGTCACAAATTTTCAACCGAGG
Phvul.002G021600	PvSnRK2.3-RT-F	AATTTCAGAAGCTACCGTACCA
	PvSnRK2.3-RT-R	CTGCTATCTACATCTTGCTCGA
Vigun06g213200	VuPR1-RT-F	ATGGGGCTATGCATTAAGGT
	VuPR1-RT-R	ATAGGGTCTTTCGCCGACAT
Vigun11g000700	VuABA3-RNAi-BamHI-F	TGGAGAGGACACGCGGGATCCAGTTAGAGAAGACAGGATTT
	VuABA3-RNAi-BamHI-R	GGCGCGCCCCATGCGGGATCCGCCAAGGGAAGTGCTACCAT
	VuABA3-RNAi-XbaI-F	TTGCAGGTATTTGGCTCTAGAGCCAAGGGAAGTGCTACCAT
	VuABA3-RNAi-XbaI-R	GGTCTTAATTAACTCTCTAGAAGTTAGAGAAGACAGGATTT
	VuABA3-RT-F	GCCAATGAAGCACAGTTC
	VuABA3-RT-R	ACCTCCAGATACCACAAGA
Vigun03g187300	VuABF2-RT-F	AACTAGGAGTGGTGAGTTGTAC
	VuABF2-RT-R	CTTCAAAGCATTGTAGTGGTCT
Vigun01g012200	VuABI5L-RT-F	ATGGTCTCACACTCAATGAAGT
	VuABI5L-RT-R	CTGCTTTCACCAAGAAATCCTC
Vigun09g114100	VuSNRK2.1-RT-F	GAGGAAGAGGAGGATGAAGAAG
	VuSNRK2.1-RT-R	AGCGATCATTGATACACGAACT
Vigun02g140800	VuSNRK2.3-RT-F	AATTTCAGAAGCTACCGTACCA
	VuSNRK2.3-RT-R	GCTCTCTAAAATGCAGACGATG
Psat1g561200	PsPR1-F	ATGAGTTCATTTTCTATATT
	PsPR1-R	ATATGGCTTCTGGCCGACAT
Psat7g228480	PsABA3-RNAi-BamHI-F	TGGAGAGGACACGCGGGATCCAGTTAGAGAATTCAGTATGT
	PsABA3-RNAi-BamHI-R	GGCGCGCCCCATGCGGGATCCGCAAAAGGATCTGCTACCAT
	PsABA3-RNAi-XbaI-F	TTGCAGGTATTTGGCTCTAGAGCAAAAGGATCTGCTACCAT
	PsABA3-RNAi-XbaI-R	GGTCTTAATTAACTCTCTAGAAGTTAGAGAATTCAGTATGT
	PsABA3-RT-F	TTGGTGCTGTAAGAGTATCC
	PsABA3-RT-R	GCTTCCATTATTGCTAAGAGG
Psat7g122760	PsABF2-RT-F	GAGACAGCCAACATTAGGT
	PsABF2-RT-R	TGAAGGAGGAAGAGGAACA
Psat4g141680	PsABI5L-RT-F	GATACTACTGCTGGAATGGA
	PsABI5L-RT-R	GTGTAACGGCTGTGCTAT
Psat5g057480	PsSNRK2.1-RT-F	GCCACAAGATTGATGAGAAC
	PsSNRK2.1-RT-R	TGAACCTTCCAGCATTACAT
Psat2g164760	PsSNRK2.3-RT-F	ATCTTCGGTGCTTCATTCA
	PsSNRK2.3-RT-R	AATACTGGACGCTGAGAAC
CPMMV-TGB1	TGB1-F	ATGAATGAACTGATCAGTAAAC
	TGB1-R	TCACTCAGAGTTTGGATAGG
CPMMV-TGB2	TGB2-F	ATGCCACTGACTCCACCACC
	TGB2-R	TCAGTGAACCCTATTGCAGA
TRV	TRV-RT-F	TCTACTTCGAACCGTGGCAG
	TRV-RT-R	CCAACTCTCGCGTTGATTCG
BBWV2	BBWV2-RT-F	TCAATTGCCAGGTAGCTCCG
	BBWV2-RT-R	TTCCACCAATCCGCACAAGA

Here, we report on the development of a molecular toolkit encompassing *in planta* transient overexpression and RNA interference, virus-induced overexpression and multi-target gene editing, all based on convenient agroinoculation. The first tool is the *Agrobacterium-*mediated gene overexpression/silencing system, which is suitable for molecular assays like protein subcellular location and bimolecular fluorescence complementation (BiFC). From screening four genotypes, 1-week-old seedlings of the common bean cv. ‘Honghuabaijia’ were found to be optimal, and provided appropriate seedling size and leaf tenderness. Two *Agrobacterium* strains (GV3101 and EHA105) carrying the PCV-GFP or PBI121-GFP constructs (four combinations in total) were tested in a negative pressure vacuum pumping method. Briefly, *Agrobacterium* was infiltrated into the abaxial surface of the leaves by negative pressure (0.08 MPa) for 3 minutes (Fig. 1a). At 60–96 hours post-infiltration (hpi), fluorescence in the infiltrated leaves was examined, which revealed widespread GFP fluorescence, with the leaves infected by GV3101 strain carrying PCV-GFP expressing the strongest signals (Fig. 1b and c). Using this optimized protocol, we tested the robustness of the system using the BiFC assay. Genes encoding two known interactive proteins, TGB1 and TGB2, from cowpea mild mottle virus (CPMMV) were cloned and inserted into the PCV-NYFP and PCV-CYFP vectors (Table 1). Three days following vacuum infiltration with GV3101 carrying TGB1-NYFP and TGB2-CYFP, yellow fluorescence was seen around the cell membrane and in the nucleus, corroborating physical interaction of the two proteins (Fig. 1d). Notably, BiFC assay of the same proteins in the *Nicotiana benthamiana* heterogeneous system revealed spotted fluorescence signals [3]. Given that CPMMV naturally infects legumes but not *N. benthamiana*, the assay in the natural host species is considered to be more biologically meaningful. This method is also highly valuable for validating gene functions involved in abiotic stress, as recently reported by Fang *et al*. [4] for a heavy-metal responsive gene, *PvXTH23*, encoding xyloglucan endo-transglycosylase (Fig. 1e). The suitability and robustness of the method was also proved in cowpea (cv. A1212, Fig. 1k, l, and m) and pea (cv. 610, Fig. 1r and s). *Agrobacterium* could be well infiltrated into cowpea and pea leaves through a syringe, and thus the protocols were more simplified and flexible in these crops.

We next demonstrated the adapted protocol of *Agrobacterium*-mediated transient gene silencing (AMTS). We selected *PvABA3*, a gene involved in the last step of abscisic acid (ABA) biosynthesis and the regulation of stomatal closure, as the target [5]. To create a stem–loop structure, a 300-bp fragment of *PvABA3* and its reverse complement fragment were amplified and inserted into the vector pFGC-5941 (Fig. 1b). Common beans were then infiltrated with *Agrobacterium* GV3101 harboring the PvABA3-RNAi or GFP-RNAi control vector. qRT–PCR showed that *PvABA3* expression at 72 hpi was downregulated to ~26% of that in the GFP-RNAi control (Fig. 1f). We observed a 1.5-fold increase in stomatal aperture in the PvABA3-RNAi-infiltrated leaves, supporting its role in negatively controlling stomatal opening. We checked the expressions of four known ABA-pathway downstream genes, including the transcription factor genes *PvABF2* and *PvABI5L* and the kinase genes *PvSNRK2.1* and *PvSNRK2.3.* Except for *PvSNRK2.3*, all genes were downregulated in the PvABA3-RNAi-infiltrated leaves (Fig. 1f), consistent with *PvABA3* interference. This system was proved to also be functional in cowpea and pea with *VuABA3* and *PsABA3* as the target gene (Table 1), respectively, demonstrating great promise for studying gene functions in legumes (Fig. 1n and t).

Nevertheless, the short duration (60–96 h) of the transient systems restricts their use in studies where prolonged gene expression is required. Thus, we adapted a tobacco rattle virus-induced overexpression (TRV-VOX) system for continuous expression of transgenes, with no need for particle bombardment or harming the normal growth of plants. We cloned *PvPR1*, a known broad-spectrum resistance gene [6] to create TRV-RNA2-PvPR1-GFP (Fig. 1b and Table 1). *Agrobacterium* GV3101 carrying TRV-RNA1/TRV-RNA2-PvPR1-GFP constructs were co-infiltrated into common bean leaves, with TRV-RNA1/TRV-RNA2-GFP as control. A devastating pathogen, broad bean wilt virus 2 (BBWV2), was inoculated at 7 days post-infiltration (dpi). After transformation, the GFP fluorescence became visible at 7 dpi and lasted at least until 15 dpi (Fig. 1g). Compared with the control, plants overexpressing *PvPR1* displayed milder disease symptoms and lower pathogenic mRNA accumulation following BBWV2 infection (Fig. 1h and i). The system was also tested in cowpea and pea by overexpressing *VuPR1* (Fig. 1o, p and q) and *PsPR1* (Fig. 1u and v), respectively, where similar enhanced resistance to BBWV2 was observed. Notably, pea plants at an older age (2 weeks) and larger size were infiltrated using a syringe. We attempted to use the TRV-based VIGS system in the legume vegetables, but failed to see positive results despite detection of TRV in the inoculated leaves. We speculate that due to the incompatibility of TRV and the silencing machineries in legumes, the production of small RNAs was inefficient.

CRISPR/Cas-mediated gene editing has been reported in cowpea and pea [7]. To date, gene editing has not been utilized in common bean, and hence we established a high efficiency, multi-target gene editing system in this species. Four sgRNAs (Fig. 1j) were designed to target exons 2, 3, and 5 of the *PvPDS* gene, encoding a phytoene desaturase, which were then simultaneously built into the multiplex knockout vector pGmUbi-Cas9-4XsgR initially designed for soybean [8]. The vector was introduced into the *Agrobacterium rhizogenes* K599 strain. 1-week-old seedlings (cv. ‘Biyuhonghua’) were infected with the transformed K599 at the hypocotyl as described [9]. Four weeks later, the induced hairy roots were collected for PCR of the marker gene *Bar*, and the *Bar* gene could be detected in all seedlings. Sequencing results of the amplified fragment showed the target sequences were edited in 11 out of the 16 plants, demonstrating a high editing efficiency of 68.75% (Fig. 1j). This result demonstrated that the pGmUbi-Cas9-4XsgR system-based multiplex gene editing works effectively in common bean, holding great promise for genetic manipulation in legume crops.

Collectively, we developed and exemplified the use of an efficient and easily operational toolkit for transformation-recalcitrant vegetable legumes, which can largely break the long-standing bottleneck of functional studies. *In planta* transient overexpression, different from leaf disk infiltration [10], provides a means of studying protein subcellular localization and interactions, which outperforms heterogeneous systems; when coupled with AMTS, it is useful for rapid validation of gene functions. The TRV-VOX system adapted for legumes, in conjugation with existing VIGS approaches, offers a solution for gene functional studies in the longer term. The multiplex gene editing system outperforms single-target gene editing and will greatly facilitate gene knockout. Future work is needed to exploit the usefulness of this toolkit in more legume crops, such as chickpea and pigeon pea, to boost their functional genomics.

## Acknowledgements

This work was supported by the National Key Research & Development Program of China (2022YFE0198000), the National Nature Science Foundation of China (32202470, 32202521), the State Key Laboratory for Managing Biotic and Chemical Threats to the Quality and Safety of Agro-products (2021 DC700024-KF202217), and the Natural Science Foundation of Zhejiang Province (LQ21C150004). We thank Professor Deyue Yu for kindly providing the pGmUbi-Cas9-4XsgR vector.

## Author contributions

X.W. and P.X. conceived and designed the experiments. P.Z., S.C., Z.Z., Y.Z., P.F., K.N., and T.S. helped to perform the experiments. P.X. helped revise the manuscript. All authors read and approved the final manuscript.

## Data availability

Data are available upon request to the corresponding author.

## Conflict of interest

The authors declare no conflicts of interests.
